# Remeex^®^ System Effectiveness in Male Patients with Stress Urinary Incontinence

**DOI:** 10.3390/jcm10102121

**Published:** 2021-05-14

**Authors:** Gerardo-Alfonso Márquez-Sánchez, Bárbara-Yolanda Padilla-Fernández, Miguel Perán-Teruel, Pedro Navalón-Verdejo, Sebastián Valverde-Martínez, Magaly-Teresa Márquez-Sánchez, Javier Flores-Fraile, María-Fernanda Lorenzo-Gómez

**Affiliations:** 1 Department of Surgery, University of Salamanca, 37007 Salamanca, Spain; gamarquezs@gmail.com (G.-A.M.-S.); sebasv_2000@hotmail.com (S.V.-M.); mflorenzogo@yahoo.es (M.-F.L.-G.); 2 Section of Urology, Department of Surgery, University of La Laguna, 38200 Tenerife, Spain; padillaf83@hotmail.com; 3 Department of Urology, University Hospital Arnau de Villanova, 46015 Valencia, Spain; mperanurologo@gmail.com; 4 Department of Urology, University Hospital Casa de Salud, Catholic University of Valencia, 46021 Valencia, Spain; pedronavalon@yahoo.com; 5 Renal Urological Multidisciplinary Research Group (GRUMUR), Institute of Biomedical Research of Salamanca (IBSAL), 37007 Salamanca, Spain; magalymarquez77@gmail.com; 6 Department of Urology, University Hospital of Ávila, 05004 Ávila, Spain; 7 Department of Urology University Hospital of Salamanca, 37007 Salamanca, Spain

**Keywords:** male stress urinary incontinence, Remeex^®^ system, slings, effectiveness

## Abstract

Background: When conservative management fails, patients with stress urinary incontinence (SUI) are considered for surgical treatment. Simpler, more economical and less invasive surgical techniques, such as the Remeex^®^ system, have been developed. Objectives: To analyze the objective effectiveness of the Remeex^®^ system in the treatment of male stress urinary incontinence. To study survival and complication rates of the Remeex^®^ system in male SUI patients. Materials and methods: Prospective observational study between July 2015 and May 2020. Group A (*n* = 7; GA) patients with mild SUI. Group B (*n* = 22; GB) patients with moderate SUI. Group C (*n* = 18; GC) patients with severe SUI. Effectiveness was assessed by the number of patients achieving complete and partial dryness. Complete dryness was defined as patients using 0–1 safety pads per day; partial dryness as a >50% reduction in the number of pads used. Results were analyzed using descriptive statistics, Student’s *t*-test. Chi2, Fisher’s exact test, ANOVA, and multivariate analysis. Significance was set at *p* < 0.05. Results: Mean age 69.76 years, mean follow-up 33.52 months. Objective effectiveness was observed in 89.36% of patients with incontinence. The effectiveness was 85.71% in GA, 90.91% in GB and 88.89% in GC. There were no significant differences among groups (*p* = 1.0000). 34.04% of patients with an implant required at least one readjustment, while 66.00% did not require any. There were no significant differences among groups (*p* = 0.113) Chi2 = 4.352. 95.74% of implants remained in place by the end of follow-up. We observed complications in 17.02% of patients. Conclusions: Remeex^®^ system is an effective and safe method for male stress urinary incontinence treatment, regardless of the severity of the incontinence, with high survival and low complication and removal rates. System readjustments are required in one-third of the cases.

## 1. Introduction

The International Continence Society defines urinary incontinence (UI) as the manifestation of any kind of involuntary loss of urine. In clinical settings, UI can be classified as stress urinary incontinence (SUI), urge urinary incontinence (UUI) or mixed urinary incontinence (MUI) [1,2].

In SUI, urine loss occurs on effort or physical exertion and is not preceded by the urge to urinate. When urine loss is associated with urge (sudden and imperious need to urinate that is hard to delay), it is considered as urge urinary incontinence (UUI), with MUI being a combination of both (stress and urge) [2,3].

UI treatment involves conservative management such as lifestyle changes, physical and behavioral therapy, as well as pharmacological treatment by means of antimuscarinics. Patients in which conservative management fails are considered for surgical treatment using external compression devices, bulking agents, urinary sphincter implants or slings, which are recommended in mild–moderate UI [4].

Artificial urinary sphincter implants (AUS) have proven to be the most successful treatment (90% success rate) but require a certain degree of cognitive capacity for the patient to operate the device, and 30–50% of patients require surgical revisions due to mechanical failure, infections or urethral atrophy [5]. This has led to the development of simpler, more economical and less invasive techniques such as adjustable sling devices. In men, three main systems have been used: the REMEEX^®^ system, the AdVance^®^ system, the ARGUS^®^ system and the ATOMS^®^ system [4].

Remeex^®^ (*REgulador MEcánico EXterno—*External Mechanical Regulator) (Neomedic©, Terrassa, Barcelona, Spain) is a device made in Spain, designed initially for the treatment of stress urinary incontinence (SUI) in women, and then introduced in 2003 for treating SUI in men [6].

### 1.1. Remeex^®^ System Description

**Figure 1:**
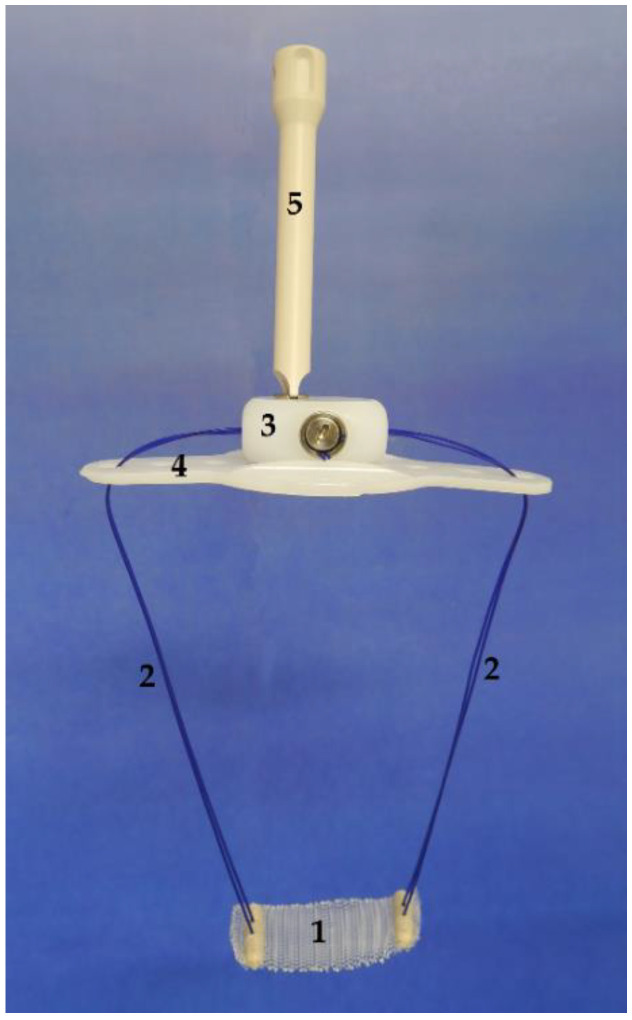
Remeex^®^ system components. Remeex^®^ system components: monofilament suburethral sling (1), traction threads (2), *varitensor* (3), *base plate* (4) and *external manipulator* (5). Courtesy of Neomedic International SL.

The system (Figure 1) comprises a polypropylene monofilament suburethral sling with two polypropylene traction threads connected to a subcutaneous regulator called a *varitensor*, which attaches to a base (placed in the suprapubic region over the abdominal rectus fascia 2 cm above the pubic symphysis) into which an *external* *manipulator* is inserted, which in turn passes through the suprapubic incision allowing for postsurgical readjustments. The *disconnector* is a specific device used to connect and disconnect the *manipulator* to the *varitensor*. Finally, the *suture passers*, consisting of a pair of retropubic, disposable needles and handle which can be disassembled, are used to guide the traction threads from the perineal to the abdominal incision during the intervention [7,8,9].

### 1.2. Surgical Protocol

System placement was conducted under regional or general anesthesia, after the administration of antibiotic prophylaxis, in a lithotomy position with moderate Trendelenburg, having shaved the abdomen and perineum as preparation. After urethral catheterization with an 18 Fr Foley Catheter, a 4–5 cm mid-line transversal suprapubic incision 2 cm above the pubis is made, dissecting until the anterior rectus muscle fascia is seen, and another vertical perineal incision of approximately 4 cm is made, dissecting subcutaneous tissue until the angle conformed by the bulbocavernosus and ischiocavernosus muscles is identified. The perineal body is dissected to mobilize the urethra.

**Figure 2:**
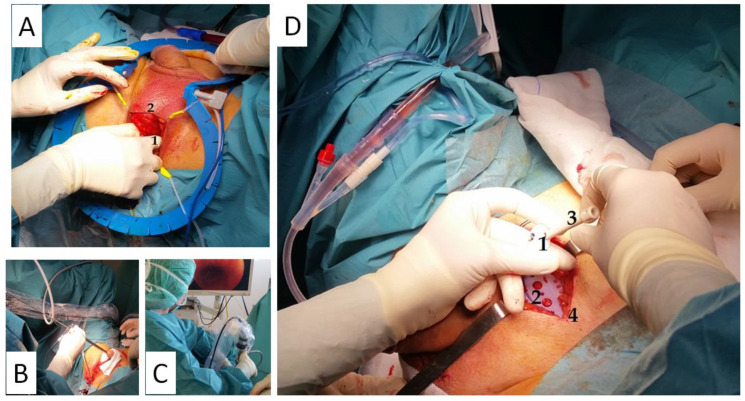
Remeex^®^ system surgery. Intraoperative images of Remeex^®^ system surgery: (**A**) = tip of the finger (1) in the newly formed retropubic space, creating a digital ascending dissection through the perineal incision (2). (**B**,**C**) = cystourethroscopy performed to confirm urethrovesical integrity. (**D**) = *varitensor* (1), *base plate* (2) and the *external manipulator* (3) connected to the sutures of the sling and placed above the aponeurosis of the rectus abdominis muscle in the suprapubic incision (4). Courtesy of Dra. María Fernanda Lorenzo Gómez, Head of the Department of Urology of University Hospital of Salamanca. Salamanca, Spain.

Subsequently, the interior edge of the ischiopubic ramus is dissected, and the urogenital diaphragm’s fascia is cut open close to the bone until it is possible to insert the tip of a finger in the newly formed retropubic space, creating a digital ascending dissection (Figure 2). One of the *suture passers* is then guided from the perineal to the suprapubic incision until the tip of the *suture passer* shows up at the abdomen. This passage is performed by placing the fingertip along the retropubic space behind the inner surface of the bone to avoid damaging the urethra or perforating the bladder. The same puncture maneuver is repeated on the contralateral side, and a cystourethroscopy is performed to confirm urethrovesical integrity (Figure 2).

Afterwards, the sutures are connected to the holes at the back of the *suture passers* and taken to the suprapubic incision, where the edge of the traction threads are clamped with mosquitoes protected with silicone tubes. The traction threads are pulled until the polypropylene sling is in contact with the bulbocavernosus muscle, ensuring that the mesh is completely extended. The sling is then fixed to the bulbocavernosus muscle using two absorbable sutures to avoid anteroposterior or lateral movement, and the perineal incision is closed using absorbable materials.

**Figure 3:**
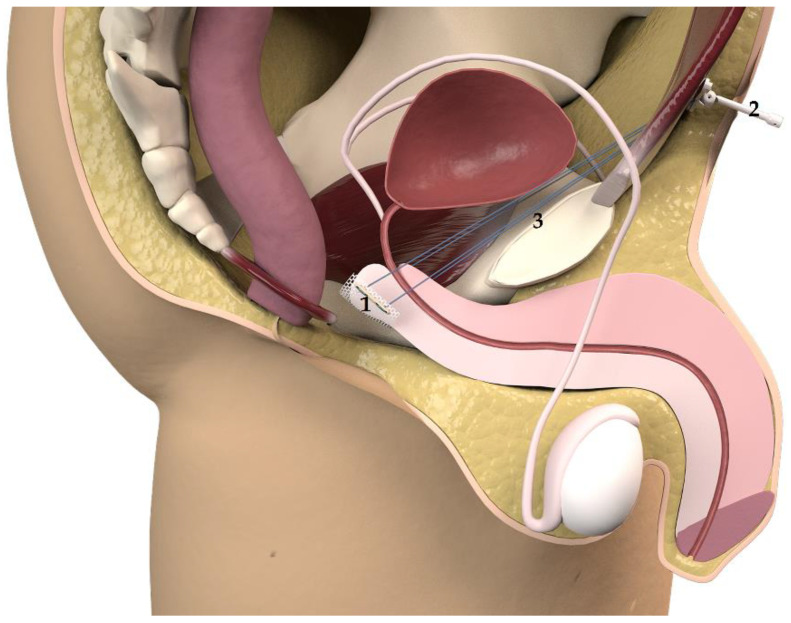
Remeex^®^ system in place. Remeex^®^ system in place after surgery: sling in contact with the bulbocavernosus muscle (1), base plate and *varitensor* and *external manipulator* (2) connected to the sutures of the sling (3) 10 cm above the aponeurosis of the rectus abdominis muscle. Courtesy of Neomedic International SL.

The *base plate* and the *varitensor* are then connected to the sutures of the sling and placed at 10 cm above the aponeurosis of the rectus abdominis muscle (Figure 2). The sutures are fixed by locking the screw in the *varitensor,* and the excess suture is cut flush. By rotating the *manipulator* clockwise, the threads are wound inside the *varitensor*. The *manipulator* must be rotated clockwise until the *varitensor* and the *base plate* rest on top of the rectus abdominis muscle fascia, where they will remain placed for future readjustments. Finally, the suprapubic incision is closed, leaving the *manipulator* out through the center of the wound (Figure 2 and Figure 3).

The first revision takes place during the immediate postoperative period (first 24–48 h). In the absence of urethral or bladder perforation, the bladder is filled with 300 cc of saline through a catheter, which is then removed. The patient is then asked to perform Valsalva maneuvers (cough) to check for urine loss. In the event of incontinence, the *manipulator* could be turned clockwise (registering the number of turns) until urine loss ceases. The patient is then asked to empty his bladder, verifying there is no postvoid residual volume, and the *manipulator* is detached using the *disconnector*.

The procedure may entail the following adverse events or complications: perforation of internal structures/nerves/blood vessels, bleeding, extrusion, erosion, surgical wound infection or dehiscence, fistulae, seromas, palpable sling, pain or discomfort, urinary tract infection (UTI), urinary retention, urge incontinence, voiding dysfunction or difficulty, urinary or intestinal obstruction, dyspareunia, dysuria or haematuria [9].

Existing evidence of the capacity to overcome UI using male sling systems is limited [4], which is the reason for conducting the present study.

The objective of this study was to analyze the objective effectiveness of the Remeex^®^ system in the treatment of male stress urinary incontinence. Additionally, to study survival and complication rates of the Remeex^®^ system in male SUI patients.

## 2. Methods

A multicenter observational prospective study was conducted at the University Hospital of Salamanca (Salamanca, Spain) and in the Department of Urology University Hospital Casa de Salud. Catholic University of Valencia. (Valencia, Spain) between July 2015 and May 2020, including 47 male patients with mild, moderate or severe stress urinary incontinence classified according to the number of pads per day (PPD). Study groups: Group A (GA): patients with mild stress urinary incontinence (1–2 PPD). Group B (GB): patients with moderate stress urinary incontinence (3–4 PPD). Group C (GC): patients with severe stress urinary incontinence (5 or more PPD). In total, 29.79% of patients received radiotherapy treatment. Additionally, 65.96% of patients had a history of open radical prostatectomy, 25.53% of laparoscopic radical prostatectomy and 8.51% had a transurethral resection of the prostate (TURP). Finally, 23.41% of patients had previous anti-incontinence surgical treatment: 19.15% had been treated with AdVance^®^ and 4.26% with ATOMS^®^ sling.

Variables: age, body mass index (BMI), follow-up time, effectiveness, complications, surgical history and number of readjustments.

Success/complete dryness or continence was defined as patients only using 0–1 safety pads per day; partial dryness as a >50% reduction in the number of pads used; treatment failure if the reduction in the number of pads was <50%. Effectiveness was measured as the number of completely and partially dry patients. Results were analyzed using descriptive statistics, Student’s *t*-test. Chi2, Fisher’s exact test, ANOVA (Scheffé’s test for normally distributed samples and Kruskal-Wallis for other distributions), and multivariate analysis. All analyses were conducted using IBM Corp. Released 2017. IBM SPSS Statistics for Windows, Version 25.0. Armonk, NY: IBM Corp.Significance was set at *p* < 0.05.

### Ethical Considerations

The study protocol 2015/2017/230/310 was approved by the Avila Healthcare University Complex Drug Research Committee (*Comité de Investigación con Medicamentos del Complejo Asistencial Universitario de Ávila)*.

## 3. Results

The mean age was 69.76 years, SD 5.20, median 71.00, range 58.00–80.00. The mean age in GA was 69.14, SD 5.75, range 59.00–74.00, mean age in GB was 70.26, SD 5.94, range 58.00–80.00, and the mean age in GC was 69.47, SD 4.28, range 61.00–76.00. No significant differences were found among groups (*p* = 0.739).

The mean BMI was 25.41 kg/m^2^, SD 2.29, median 25.65, range 20.20–30.12. The mean BMI in GA was 25.42, SD 2.93, range 20.55–29.74; in GB, it was 25.14, SD 2.57, range 20.20–30.12, while in GC, the mean BMI was 25.73, SD 1.69, range 21.60–28.38. No significant differences were found among groups (*p* = 0.535).

### 3.1. Follow-Up

The mean follow-up was 33.52 months, SD 10.70, median 27.68, range 21.37–57.76. The group with the longest mean follow-up period was GA with 35.18 months, which, when compared with GB, 33.18 months, and GC, 33.22 months, showed no significant differences (*p* = 0.884).

### 3.2. Effectiveness

**Figure 4:**
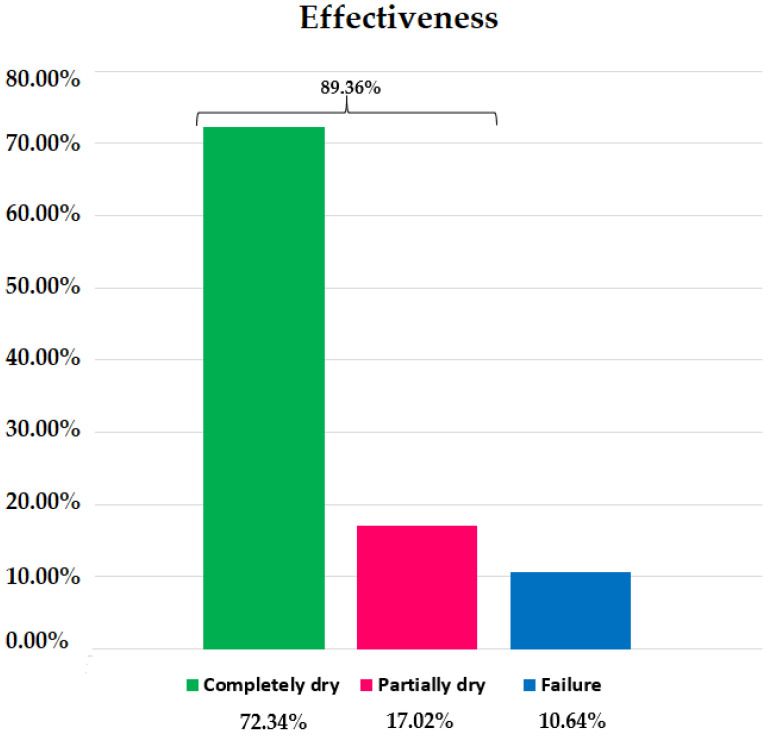
The effectiveness of the Remeex^®^ implant within the sample. Effectiveness = completely and partially dry patients.

Incontinent patients treated using the Remeex^®^ system reported 89.36% objective effectiveness (Figure 4). The group with the highest reported effectiveness within the group was GB with 90.91%, compared to 85.71% in GA and 88.89% in GC. No significant differences were found among groups (*p* = 1.0000) (Table 1), Chi-square: 0.158.

**Figure 5:**
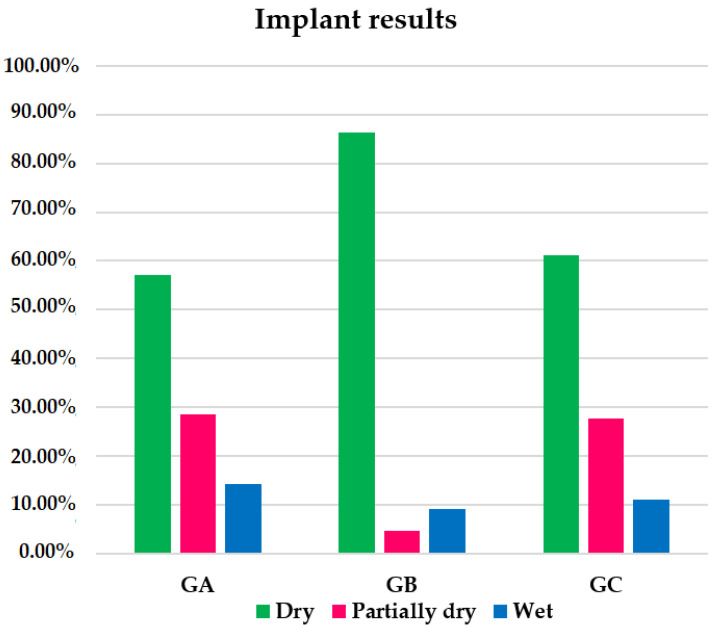
Implant results within the groups. GA: males with mild stress urinary incontinence. GB: males with moderate stress urinary incontinence. GC: males with severe stress urinary incontinence.

After the Remeex^®^ system implant, 72.34% of patients in the global sample were completely dry; 17.02% were partially dry; 10.64% were failed treatments/wet patients. The group with the highest percentage of completely dry patients was GB with 86.36% compared to 57.14% in GA and 61.11% in GC (Figure 5). There were no significant differences when comparing completely dry patients between GA and GB (*p* = 0.1315), GA and GC (*p* = 1.0000) or GB and GC (*p* = 0.1401) (Table 1).

The group with the highest percentage of partially dry patients within the group was GA with 28.57%, followed by GC with 27.78% and GB with 4.55% (Figure 5). There were no significant differences when comparing GA with GB (*p* = 0.136), GA with GC (*p* = 1.0000) or GB with GC (*p* = 0.0734) (Table 1).

The group with the highest percentage of treatment failures was GA (14.29%) (Figure 5). When comparing this percentage with those of GB (9.09%) and GC (11.11%) and those of GB with GC, there were no significant differences (Table 1).

### 3.3. Readjustment

In our sample, 65.96% of the patients did not receive readjustments in their Remeex^®^ system. In total, 34.04% did require readjusting; 17.02% of which received one readjustment, 14.89% received two readjustments, and 2.13% received three readjustments.

Analyzing readjustments within each group, GC was the group with the highest readjustment percentage with 44.44%, as opposed to 42.86% in GA and 22.73% in GB, while GB was the group with the highest percentage without readjustments with 77.27%, followed by 57.14% and 55.56% of groups GA and GC, respectively. GC had the highest percentage of patients with one or two readjustments (22.22%). The only patient with three readjustments belonged to GA. No significant differences were found when comparing groups, Chi-square: 8.132 (Table 2).

### 3.4. Implant Survival

**Figure 6:**
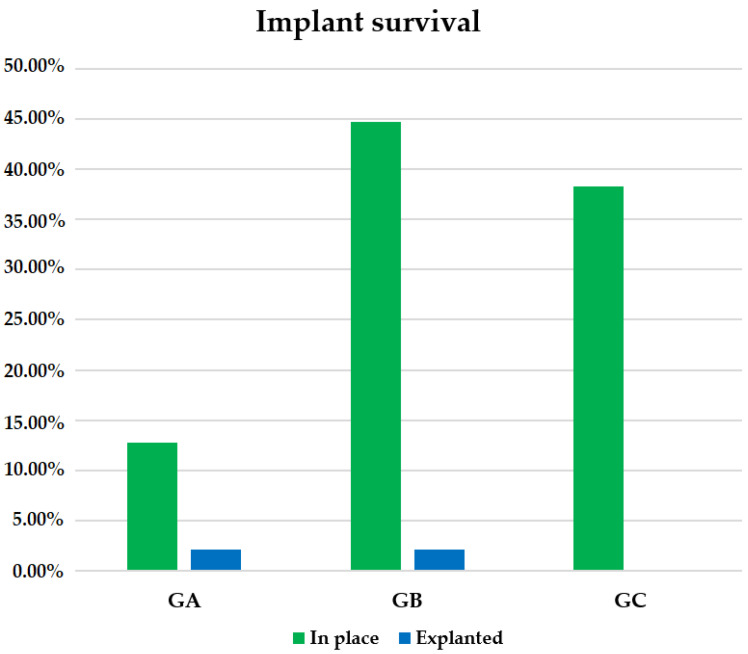
Implant survival in the sample. GA: males with mild stress urinary incontinence; GB: males with moderate stress urinary incontinence; GC: males with severe stress urinary incontinence.

In our sample, 95.74% of Remeex^®^ implants remained in place at the end of follow-up, while 4.26% had to be removed, corresponding to a total of two patients, one in GA and another in GB. In GC, 100.00% of patients maintained their implant at the end of follow-up as opposed to GA with a survival rate of 85.71% and GB with 95.45% (Figure 6). No significant differences were found among groups, Chi-square: 2.533 (Table 1).

### 3.5. Complications

A total of 17.02% of patients had a complication. Intraoperative complications happened in five patients (10.64%) in the form of bladder perforation during the insertion of the guiding needles (Table 3). In these cases, the needles were removed and reinserted to finish the implantation of the system.

Postoperative complications occurred in three patients (6.38%), and one patient in GB, which is 2.13% of the sample, reported perineal pain referred to the right posterior scrotum during implant placement but retained the implant and achieved continence despite that. One of the patients (2.13%) in GA required the removal of the system due to an infection, while the other case of removal was a patient (2.13%) in GB, with a history of radical prostatectomy and radiotherapy treatment, who developed urinary retention after Remeex^®^ implant placement (Table 3). In this last case, a urethroscopy was performed showing no evidence of urethral stenosis, so a urethrography was conducted with which bilateral vesicoureteral reflux was evidenced. The injection of bulking substances was unsuccessful, and given the options of voiding difficulty/dysfunction or urinary incontinence, we opted for the latter. No significant differences were found among groups (Table 4).

### 3.6. Surgical History

In total, 65.96% of the patients had a history of open radical prostatectomy, 25.53% of laparoscopic radical prostatectomy and 8.51% had a transurethral resection of the prostate (TURP). 23.41% of patients had previous anti-incontinence surgical treatment: 19.15% had been treated with AdVance^®^ and 4.26% with ATOMS^®^ sling. (Table 5).

### 3.7. Radiotherapy

Globally, 70.21% of patients had not received radiotherapy. Out of the 29.79% who did receive it, 14.90% belonged to group GC, 10.63% to GB and 4.26% to GA. Patients with radiotherapy history had an effectiveness of 85.71% compared to those without radiotherapy with 90.91% of effectiveness (*p* = 0.0001).

### 3.8. Multiple Regression

**Figure 7:**
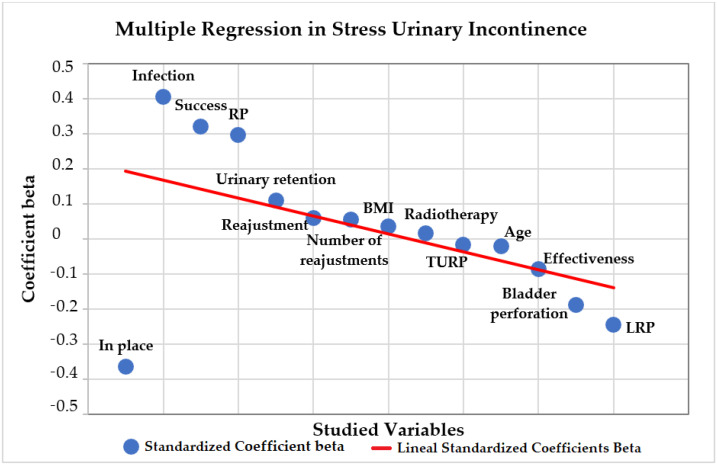
Multiple regression on the grade of stress urinary incontinence.

A multiple regression analysis was conducted on the association of the variables with urinary incontinence severity, shown in Figure 7. The direct positive association between the grade of incontinence and the variables BMI, success, readjustment, number of readjustments, radiotherapy, infection and urinary retention was not significant (Table 6). The direct negative association between age, effectiveness, in place implants and bladder perforation was not significant (Table 6). Regarding the previous oncologic surgery, RP had a direct positive association with the grade of SUI, which was significant (*p* = 0.043). LRP and TURP had a direct negative association, but was not significant (*p* = 0.299, *p* = 0.963).

### 3.9. Cox Regression Analysis

**Figure 8:**
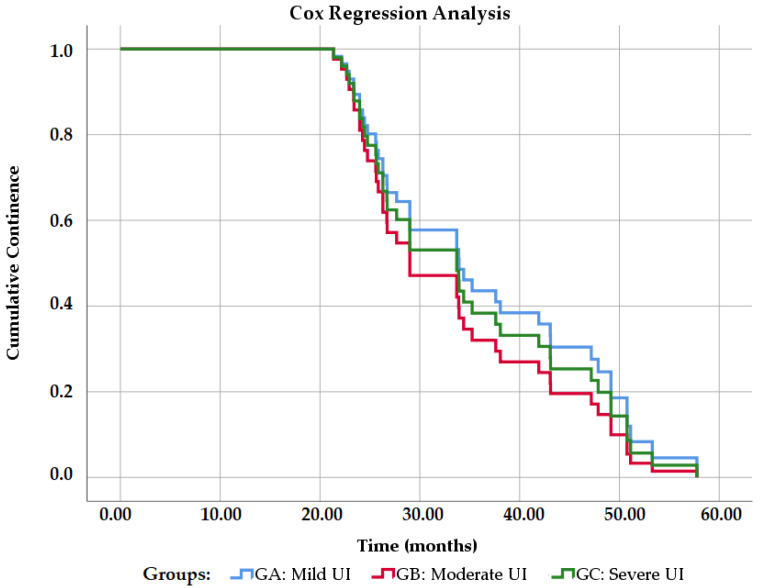
Cox regression analysis of continence probability between groups. UI: Urinary Incontinence.

The percentage of continence was 89.36% within the general sample. Figure 8 shows the probability of total continence in the study groups (degrees of urinary incontinence). Grade 2 urinary incontinence has a worse prognosis at a magnitude of 1.18. That is, they have 1.18 times more probability than the other groups of not having continence (Exp B = 1.188, *p* = 0.627). Grade 1 urinary incontinence has the best prognosis in a magnitude of 0.867 (Exp B = 0.867, *p* = 0.770), while grade 3 has a magnitude of 0.863 (Exp B = 0.863; *p* = 0.767), that is, they are 0.867 and 0.863 times more likely to not have continence, respectively.

## 4. Discussion

Urinary incontinence (UI) is a common condition worldwide, generating an important burden for people and societies, added to the uneasiness and embarrassment that it entails, which in turn reduces the quality of life of patients affected by any kind of UI [4,10,11].

When conservative management fails, patients with SUI are considered for surgical treatment, which comprises a variety of procedures, with adjustable sling implants such as the Remeex^®^ system to be considered as some of the simplest, most economical and less invasive alternatives to the artificial urinary sphincter procedure (AUS) [4]. Despite this, current evidence on the effectiveness of male slings for SUI is limited, which motivated carrying out this study to provide some clarity on the matter.

Our results show that our sample comprised male patients with a mean age of 69.76 years, which was also observed in previous studies where the mean age was between 67–70.4 years [6,7,8,12,13,14].

Follow-up for our patients ranged from 21.37 to 57.76 months with a mean of 33.52 months, similar to the follow-up period of previous studies such as the ones conducted by Sousa-Escandón et al. [6] and Leizour et al. [14], and slightly shorter than in the studies by Navalón-Monllor et al. [8] and Kim et al. [13]. Nevertheless, our minimum follow-up (21.37 months) was longer than in previous studies.

A mean BMI of 25.41 kg/m^2^ is similar to the one reported by Leizour et al. in a previous study of 26.7 kg/m^2^ [14] where this variable, similarly to our study, did not show a significant correlation with the grade of incontinence severity or the success rate of the Remeex^®^ system.

We observed effectiveness of 89.36%, i.e., out of 47 patients included in our study. The heterogenicity in the study population allowed us to determine the effectiveness of the Remeex^®^ system regardless of the severity of the SUI, as was done by Sousa-Escandón et al. [6], Kim et al. [13] and Leizour et al. [14]. In total, 42 obtained complete dryness (use of 0–1 safety pads) or an improvement with the system (>50% reduction in the number of pads used), one of the highest success rates compared to previous studies [6,12,13,14] and surpassed only by those reported by Navalón et al. in 2010 [7] and by Navalón-Monllor et al. in 2016 [8]. It is worth highlighting that, in the aforementioned studies, all patients suffered from severe SUI, which makes our study the one to report the highest effectiveness to date in patients with SUI of any given severity. This allowed us to determine if the grade of urinary incontinence had a significant effect on the effectiveness of male SUI treatment using the Remeex^®^ system. We observed that the direct negative association between the grade of incontinence and effectiveness was not significant in our multiple regression analysis (*p* = 0.810), which indicates the Remeex^®^ system is effective, regardless of SUI severity. In our Cox regression analysis, we observed that between the groups, GB (moderate SUI) had the worse prognosis with 1.18 times more probability than the other groups of not having continence (Exp B = 1.188). However, this was not significant (*p* = 0.627).

Though the effectiveness of or results is similar to previous studies [6,12,13,14], in said studies, the rate of complete dry patients (0–1 safety pads) varies from 36%–64.7% compared to the 72.34% found in our study. This could be due to the fact that, as years go by, the technique of the surgical procedure has been further standardized, allowing for better results. This could also explain the lower percentage of patients who required one or more readjustments (34.04% in our study) as opposed to previous studies where the rate varied between 60% and 91% [6,12,13,14].

As for the number of readjustments, we found the most frequent was one readjustment, similar to what was reported in previous studies [6,7,8,12,13,14], and even though readjustments were more frequent in the group with more severe incontinence (GC), the association between incontinence severity and readjustments was not significant in our multiple regression analysis (*p* = 0.436). Additionally, if we exclude the two patients (4.26%) with explanted systems, there were, between the partially dry and failure group, one patient with no readjustments (2.13%), five patients with only one readjustment (10.63%) and five patients (10.63%) with two readjustments, meaning a total of 23.40% of patients may have improved if they were given an additional readjustment, which did not happen either because the patient was already satisfied with the results or because the patient did not agree to more readjustments.

Other aspects to consider in SUI treatment using slings are the removal and complications of the system. A total of 95.74% of implants in our study remained in place at the end of follow-up, meaning only 4.26% of patients required an explant, a rate similar to the one found in more recent studies by Leizour et al. [14] and Kim et al. [13] in 2016, and slightly lower to the one found in initial studies using this system [6,8,12]. Bladder perforation during surgery was the most frequent complication (10.64%). This circumstance can be expected during the implantation of the system and, following protocol, the needles were removed and reinserted to finalize the surgical procedure, allowing for the final implantation of the system in all cases. We also observed one case of surgical site infection, one case of pain and one case of acute urinary retention, which were, along with the bladder perforation, the most frequent complications reported in other studies [6,12,13,14]. The previously mentioned were the only complications we observed out of the list of possibilities that also included bleeding, extrusion, erosion, surgical wound dehiscence, fistulae, seromas, palpable sling, urinary tract infection (UTI), urge incontinence, urinary or intestinal obstruction, dyspareunia, dysuria or hematuria, which were not observed in our study.

Patients with a history of radiotherapy had an 85.71% effectiveness compared to those without radiotherapy with 90.91% (*p* = 0.0001), which could indicate radiotherapy is related to worse results with the Remeex^®^ system as it was already pointed out by previous studies [6,13]. Regarding the previous oncologic surgery, in our multiple variable analysis, RP was directly associated with the grade of SUI, which was significant (*p* = 0.043). Meanwhile, LRP and TURP had a direct negative association (not significant *p* = 0.299, *p* = 0.963, respectively), meaning patients with RP had a more severe incontinence grade than others. This implies that the rate of incontinence is related to the type of oncological surgery performed, which has already been stated by Manfredi et al. [15] and Checcucci et al. [16].

Our results reinforce previously published studies, with the Remeex^®^ system proving to be an effective and safe solution for stress urinary incontinence treatment, even though sample sizes, follow-up periods and target population vary among studies.

## 5. Conclusions

Remeex^®^ system implants are an effective and safe method for male stress urinary incontinence treatment, regardless of the severity of the incontinence, with high survival (95.70%) and low removal (4.30%) rates. System readjustments are required in one-third of cases.

## Figures and Tables

**Table 1:** Implant results.

Implant Result	Group	Frequency	% within the Group	Group	Frequency	% within the Group	*p*-Value
Successful/dry	GA, *n* = 7	4	57.14	GB, *n* = 22	19	86.36	0.1315
	GA, *n* = 7	4	57.14	GC, *n* = 18	11	61.11	1.0000
	GB, *n* = 22	19	86.36	GC, *n* = 18	11	61.11	0.1401
Partially dry	GA, *n* = 7	2	28.57	GB, *n* = 22	1	4.55	0.136
	GA, *n* = 7	2	28.57	GC, *n* = 18	5	27.78	1.0000
	GB, *n* = 22	1	4.55	GC, *n* = 18	5	27.78	0.0734
Effectiveness	GA, *n* = 7	6	85.71	GB, *n* = 22	20	90.91	1.0000
	GA, *n* = 7	6	85.71	GC, *n* = 18	16	88.89	1.0000
	GB, *n* = 22	20	90.91	GC, *n* = 18	16	88.89	1.0000
Failure/wet	GA, *n* = 7	1	14.29	GB, *n* = 22	2	9.09	1.0000
	GA, *n* = 7	1	14.29	GC, *n* = 18	2	11.11	1.0000
	GB, *n* = 22	2	9.09	GC, *n* = 18	2	11.11	1.0000
In place	GA, *n* = 7	6	85.71	GB, *n* = 22	21	95.45	0.431
	GA, *n* = 7	6	85.71	GC, *n* = 18	18	100.00	0.2800
	GB, *n* = 18	21	95.45	GC, *n* = 18	18	100.00	1.0000
Explanted	GA, *n* = 7	1	14.29	GB, *n* = 22	1	4.55	0.431

GA: males with mild stress urinary incontinence; GB: males with moderate stress urinary incontinence; GC: males with severe stress urinary incontinence.

**Table 2:** Comparison of the number of implant readjustments.

Number of Readjustments	Group	Frequency	% within the Group	Group	Frequency	% within the Group	*p*-Value
0	GA, *n* = 7	4	57.14	GB, *n* = 22	17	77.27	0.3568
	GA, *n* = 7	4	57.14	GC, *n* = 18	10	55.56	1.0000
	GB, *n* = 22	17	77.27	GC, *n* = 18	10	55.56	1.0000
1	GA, *n* = 7	1	14.285	GB, *n* = 22	3	13.64	1.0000
	GA, *n* = 7	1	14.285	GC, *n* = 18	4	22.22	1.0000
	GB, *n* = 22	3	13.64	GC, *n* = 18	4	22.22	0.6798
2	GA, *n* = 7	1	14.285	GB, *n* = 22	2	9.09	1.0000
	GA, *n* = 7	1	14.285	GC, *n* = 18	4	22.22	1.0000
	GB, *n* = 22	2	9.09	GC, *n* = 18	4	22.22	0.381
3	GA, *n* = 7	1	14.285	GB, *n* = 22	0	0	0.0001
	GA, *n* = 7	1	14.285	GC, *n* = 18	0	0	0.0001

GA: males with mild stress urinary incontinence; GB: males with moderate stress urinary incontinence; GC: males with severe stress urinary incontinence.

**Table 3:** Complications.

Complications						
**Intra-operative**	***n***			**%**		
Uneventful bladder perforation	5			10.64%		
	**Clavien–Dindo**					
	**I**		**II**		**III**	
**Post-operative**	***n***	**%**	***n***	**%**	***n***	**%**
Perineal Pain	1	2.13				
Infection (Explantation)					1	2.13
Urinary retention (Explantation)					1	2.13

**Table 4:** Comparison of complications between the groups.

Complication	Group	Frequency	% within the Group	Group	Frequency	% within the Group	*p*-Value
Bladder perforation	GA, *n* = 7	1	14.28	GB, *n* = 22	2	9.09	0.5497
	GA, *n* = 7	1	14.28	GC, *n* = 18	2	11.11	1.0000
	GB, *n* = 22	2	9.09	GC, *n* = 18	2	11.11	1.0000
Perineal pain	GA, *n* = 7	0	0	GB, *n* = 22	1	4.54	1.0000
	GA, *n* = 7	0	0	GC, *n* = 18	0	0	0.0001
	GB, *n* = 22	1	4.54	GC, *n* = 18	0	0	1.0000
Infection	GA, *n* = 7	1	14.28	GB, *n* = 22	0	0	0.2414
	GA, *n* = 7	1	14.28	GC, *n* = 18	0	0	0.2800
	GB, *n* = 22	0	0	GC, *n* = 18	0	0	1.0000
Urinary retention	GA, *n* = 7	0	0	GB, *n* = 22	1	4.54	1.0000
	GA, *n* = 7	0	0	GC, *n* = 18	0	0	1.0000
	GB, *n* = 22	1	4.54	GC, *n* = 18	0	0	1.0000

GA: males with mild stress urinary incontinence; GB: males with moderate stress urinary incontinence; GC: males with severe stress urinary incontinence.

**Table 5:** Surgical history.

**Previous Oncologic Surgery**		
**Type of surgery**	***n***	**%**
Open Radical Prostatectomy (RP)	31	65.96
Laparoscopic Radical Prostatectomy (LRP)	12	25.53
TURP	4	8.51
Total	47	100.00
**Previous Anti-Incontinence Surgical Treatment**		
**Type of surgery**	***n***	**%**
AdVance	9	19.15
ATOMS	2	4.26
Total	11	23.41

**Table 6:** Multiple regression of variables on a global sample.

Variables	Standardized Coefficients	*p*-Value	95.0% Confidence Interval for B	
	Beta		Lower Bound	Upper Bound
(Constant)		0.734	−7.641	5.439
Age	−0.020	0.917	−0.056	0.050
BMI	0.037	0.874	−0.146	0.171
Effectiveness	−0.086	0.805	−1.751	1.370
Success	0.321	0.480	−0.612	1.273
Readjustment	0.061	0.870	−1.000	1.176
Number of readjustments	0.056	0.866	−0.519	0.613
In place	−0.363	0.101	−2.699	0.253
Radiotherapy	0.016	0.930	−0.523	0.570
Bladder perforation	−0.187	0.472	−1.572	0.746
Infection	0.406	0.071	−0.173	3.995
Urinary retention	0.110	0.571	−1.327	2.365
RP: radical prostatectomy	0.296	0.043	0.015	0.923
LRP: Laparoscopic radical prostatectomy	−0.245	0.299	−0.715	0.225
TURP: transurethral resection	−0.017	0.963	−0.761	0.726

## Data Availability

The data presented in this study are available on request from the corresponding author. The data are not publicly available due to patient data protection.
